# VHL Gene Restoration Supports RCC Reprogramming to iPSCs but Does Not Ensure Line Stability

**DOI:** 10.3390/cancers17223693

**Published:** 2025-11-18

**Authors:** Zsuzsanna Lichner, Yasaman Shamshirgaran, Katarzyna Pieczonka, Anna Jonebring, Mark Kibschull, Oksana Shynlova, Jalna Meens, Raymond H. Kim, Laurie Ailles, Bilada Bilican, Ryan Hicks, Ian M. Rogers

**Affiliations:** 1 Lunenfeld Tanenbaum Research Institute, Mt. Sinai Hospital, Toronto, ON M5G 1X5, Canada; lichner_zsuzsi@yahoo.com (Z.L.); kibschull@lunenfeld.ca (M.K.); shynlova@lunenfeld.ca (O.S.); 2 Unity Health, Toronto, ON M5B 1W8, Canada; 3 Translational Genomics, Discovery Sciences, BioPharmaceuticals R&D, AstraZeneca, 43183 Gothenburg, Sweden; yas.shamshirgaran@gmail.com (Y.S.); anna.jonebring@gmail.com (A.J.); bilada.bilican@astrazeneca.com (B.B.); 117ryanhicks@gmail.com (R.H.); 4 Division of Genetics and Development, Krembil Research Institute, University Health Network, Toronto, ON M5T 0S8, Canada; k.pieczonka@mail.utoronto.ca; 5 Institute of Medical Sciences, University of Toronto, Toronto, ON M5S 3H2, Canada; 6 Department of Physiology, University of Toronto, Toronto, ON M5S 1A1, Canada; 7 Princess Margaret Cancer Centre, Cancer Clinical Research Unit (CCRU), Toronto, ON M5G 1Z5, Canada; jalna.meens@uhnresearch.ca (J.M.); raymond.kim@uhn.ca (R.H.K.); 8 Department of Medical Biophysics, University of Toronto, Toronto, ON M5G 2C4, Canada; laurie.ailles@uhnresearch.ca; 9 Princess Margaret Cancer Centre, University Health Network, Toronto, ON M5G 2C4, Canada; 10 School of Cardiovascular and Metabolic Medicine, King’s College London, Strand, London WC2R 2LS, UK; 11 Department of Obstetrics and Gynecology, University of Toronto, Toronto, ON M5G 1E2, Canada

**Keywords:** clear cell renal cell carcinoma, reprogramming, VHL, oxidative phosphorylation, glycolytic, cancer cell-iPSC, cancer model

## Abstract

Background: Modeling precancerous stages holds the promise to understand early transformation events, thereby offering the potential for personalized, targeted treatment. Because cancer hijacks developmental pathways, precancerous stages could potentially be modeled by reprogramming cancer cells to an induced pluripotent stem cell state and subsequently differentiating them to the target organs using organoid models. Methods: We attempted reprogramming of patient-derived clear cell renal cell carcinoma (ccRCC) cell lines and adjacent normal renal epithelial cell lines using lentivirus or episomal reprogramming vectors. Results: The cancer cells failed to reprogram while the adjacent normal cells reprogrammed with high efficiency. The von Hippel–Lindau factor (VHL) gene was re-expressed in ccRCC cells in an attempt to restore the wild-type phenotype and restore reprogramming. The VHL gene is the major tumor suppressor in ccRCC pathogenesis and a conductor of oxidative-glycolytic glucose metabolism. While its re-expression did restore the epithelial phenotype and oxidative regulation of ccRCC cells, they still failed to stably reprogram. With an optimized reprogramming workflow, VHL-corrected ccRCC cells generate NANOG+ cells; however, they remained dependent on the ectopic expression of the reprogramming factors. Conclusions: We concluded that while VHL expression is necessary for cellular reprogramming of ccRCC cells, other genetic lesions in the ccRCC cells could be preventing the stabilization of the pluripotent state.

## Simple Summary

The von Hippel–Lindau Syndrome, an inherited condition that predisposes to clear cell renal cell carcinoma (ccRCC), affects developmental pathways of the kidney. Studying these early stages could help develop customized, targeted treatments. One approach is to reverse-engineer cancer cells into a stem cell-like state and then grow them into organ models. We attempted this with clear cell renal cell carcinoma (ccRCC) cells and adjacent normal (AN) healthy kidney cells using cell reprogramming. While the adjacent normal cells successfully reprogrammed to induce pluripotent stem cells (iPSCs), the cancer cells did not. We then restored the VHL gene—often missing in ccRCC. This reintroduced normal cell characteristics, but the cells upon reprogramming could not maintain a stable stem cell state. Our findings suggest that while restoring VHL is necessary for reprogramming, other genetic issues block ccRCC cells from fully returning to a healthy, stem-like state, indicating the importance of other, yet to be determined factors. Similarities between cancer cells and stem cells have been recognized, as common genes can influence both outcomes. In this study, we demonstrate that the VHL gene can be added to this group, as it is involved in both cancer development and stem cell regulation.

## 1. Introduction

There were 73,000 incidences and 14,000 deaths in the United States in 2022 due to renal cell carcinoma (RCC) [1,2]. Clear cell renal cell carcinoma (ccRCC) is the most relevant clinical subtype, due to its high frequency (44% of all kidney tumors), and ccRCC accounts for 34% of deaths. Multiregional next-generation sequencing studies indicate a high intra-tumoral genetic heterogeneity which may explain the poor drug response and frequent treatment resistance [3,4]. A potential solution is to focus therapeutic efforts on the premalignant lesions, a stage with low genetic diversity; however, this stage remains largely uncharacterized due to the lack of an appropriate model. The von Hippel–Lindau Syndrome (VHLS) is a rare, autosomal dominant, inherited form of ccRCC. VHLS patients have early-onset tumors and can develop multiple recurring ccRCC tumors, resulting in a poor prognosis [5]. Genetic studies, next-generation sequencing data and transgenic mouse models illustrate the central role of the von Hippel–Lindau (VHL) tumor suppressor and the downstream hypoxia pathway in ccRCC pathogenesis [6,7,8,9]. Approximately 70% of VHLS patients develop malignant ccRCC by the age of sixty, accompanied by the loss of the second WT VHL allele. VHL is an E3 ubiquitin ligase that recognizes the Hypoxia-Induced Factors (HIFs) and marks them for proteasomal degradation [10]. Clear cell renal cell carcinoma (ccRCC) tumors frequently display both hemizygous and heterozygous genetic changes. A defining feature, however, is loss of heterozygosity (LOH) resulting from hemizygous deletions, most notably involving the VHL gene on chromosome 3p. Whether alterations appear as hemizygous or heterozygous depends on the underlying chromosomal event: tumors with whole-arm deletions typically show hemizygosity, whereas retention of both alleles—one of which carries a mutation—produces heterozygosity.

New disease models are required for ccRCC in order to develop improved therapies. ccRCC premalignant stage is technically difficult to observe, and transgenic mouse models and xenograft models seem to advance to a high-grade tumor without reproducing the initial histopathological changes. Based on the co-existence of multifocal ccRCC lesions and renal cysts in the von Hippel–Lindau syndrome patients, the current hypothesis is that the dilation of renal epithelial tubules gives rise to cystic structures that eventually transform to ccRCC. However, direct observation of the proposed premalignant structures and their progression has not been reported due to technical challenges. A promising solution is to model early oncogenic events in vitro by reprogramming cancer cells into induced pluripotent stem cells (iPSCs) and subsequently re-differentiate them into the organ-of-origin of the primary cancer. Kidney cancer tumorigenesis relies on reactivation of developmental pathways, particularly those governing simple kidney structures, such as the tubules [11,12,13,14,15]. Notably, precursor lesions of clear cell renal cell carcinoma are thought to originate from epithelial tubules [16,17]. We propose that by driving ccRCC-iPS cells through morphogenesis, divergence from normal tissue differentiation could capture key events in cancer initiation. Using this workflow, Kim et al. was able to generate iPSC lines from pancreatic intraepithelial neoplasia lesions, the precursor of pancreatic ductal adenocarcinoma [18]. iPSC lines could also be generated from several hematological malignancies [19,20]. However, success seems to be tissue and mutation dependent. In a recent review of adult cell and pluripotent cell-derived organoids for renal cancer modeling, only adult cell-derived kidney spheroids were successful, while derivation of kidney cancer-model organoids from pluripotent cells was not [21]. It is now widely recognized that reprogramming is a significant bottleneck in developing iPSC-based cancer models [22,23,24]. A possible explanation is that cancer driver mutations interfere with somatic cell reprogramming. Here we study the role of the VHL gene in reprogramming ccRCC.

The VHL/HIF axis is a master regulator of glycolytic and oxidative metabolic balance of the cell. VHL loss and uncontrolled HIF activity contribute to the metabolic rewiring of cancer cells by enhancing the glycolytic flux and inhibiting oxidative phosphorylation (OXPHOS) [25,26,27]. In addition, metabolic rewiring in cancer cells is closely tied to the regulation of heme-dependent enzymes. Recent studies have shown that GAPDH and Hsp90 act as critical chaperones for intracellular heme transport and allocation to diverse heme proteins, including NO synthases and dioxygenases, thereby directly influencing mitochondrial respiration and redox balance [28]. Importantly, somatic cell reprogramming is initiated by a burst of oxidative phosphorylation (OXPHOS) activity followed by a metabolic shift from the OXPHOS-dependent terminally differentiated cells to the glycolytic pluripotent state. Without the OXPHOS burst there can be no initiation of reprogramming. This produces a reprogramming block for the glycolytic cancer cells. The reason some cancer cells are amenable to reprogramming is that despite the initial findings and assumption that all cancer cells are in the glycolytic state, it has been well documented that some cancer types do use the OXPHOS pathway for respiration, making them more amenable to reprogramming [29]. Both ccRCC cells and pluripotent stem cells rely on HIF1α expression, a key regulator of hypoxia response, leading to the hypothesis that the dysregulation of the VHL gene and the downstream glucose oxidation state is a crucial controller of ccRCC cells’ reprogramming capacity. We hypothesize that the loss of VHL in ccRCC cells affects reprogramming. In the present study we examine the reprogramming efficiency of a patient-derived ccRCC cell line and the same line with the VHL gene restored. We used a VHL mutant ccRCC line as this form of ccRCC exhibits a relatively simple, linear genetic evolution [30]. We aim to determine role of the VHL gene on reprogramming in order to establish critical pre-malignant ccRCC models.

## 2. Materials and Methods

### 2.1. Institutional Review Board Statement

The study was conducted in accordance with the Declaration of Helsinki and approved by the Institutional Review Board (or Ethics Committee) of UHN/Princess Margaret Hospital, Toronto, Canada and LTRI, Sinai Health System, Toronto, ON, Canada. REB 15-9559, REB 13-0346-E, REB 24-0044-E, REB 15-0138-E.

### 2.2. ccRCC/AN Cell Cultures

Patient samples are referred to as AN# for adjacent normal tissue and RCC# for ccRCC tissue. Radical nephrectomy specimens from a VHL syndrome tumor (RCC2) and a sporadic tumor (RCC3) were sampled along with their adjacent normal renal cortex tissue (AN2 and AN3), and cell lines were established. This provided us with cell line pairs consisting of healthy and cancerous tissue from the same patient. The surgeon obtaining the tissue is skilled in keeping the two tissues separate with no cross-contamination. An experienced pathologist confirmed the tissues as being ‘cancerous’ or ‘healthy’. Pairs are as follows: pair (1), RCC2 and AN2, and pair (2), RCC3 and AN3. AN/ccRCC cells were cultured on rat-tail collagen IV matrix (5 µg/cm^2^ BD Biosciences) in DMEM media, supplemented with 10% FBS, penicillin and streptomycin, at 37 °C and 5% CO_2_ conditions. The oxygen level in the normoxic incubator was 21% O_2_,and in the hypoxic incubator it was 3% O_2_. Cell cultures were monitored for the presence of Mycoplasma and cell identity was verified by STR profiling (TCGA Genetic Analysis Facility, The Hospital for Sick Children, Toronto, ON, Canada).

### 2.3. Cell Reprogramming

Episomal Reprogramming was used for the initial reprogramming attempts. The neon transfection system (ThermoFisher, Waltham, MA, USA) was used along with the EpiV system (Thermofisher) [31,32]. It comes with 24 different preset electroporation settings consisting of pulse number, amplitude and length. Optimization experiments were performed using a vector containing the GFP gene. Final optimized electroporation parameters were chosen for each cell sample as follows:

(i) AAVS1 VHL: RCC cells: 500,000 cells/1600 v/30 ms/1 pulse
(ii) VHL: HEK cells: 250,000 cells/1100 v/20 ms/2 pulse
(iii) RCC2: 500,000 cells/1200 v/30 ms/1 pulse
(iv) Urine-derived cells (Control): 5000,000 cells: 1400 V, pulse width 20 ms, two pulses.

One day prior to transfection cells were cultured in Penstrep free media until they were 75–90% confluent. The cells were harvested with 1.5 mL accutase and neutralized with 10 mL of DMEM + 10% FBS. 100,000 cells were resuspended in PBS(−/−) and centrifuged at 400× *g* for 5 min. The PBS(−/−) was then aspirated off and the cell pellet was resuspended in 100 uL of R buffer with 1 µL Epi5 vectors and 1 µL Epi5 p53& EBNA vectors.

The cells and vector mixture were then electroporated and plated onto a 10 cm geltrex coated plate with Irvine DMEM/10% FBS media and incubated for 24 h. Day 1 post transfection the cells were switched to the N2B27 media which contained DMEM/F12 with HEPES (Invitrogen, cat# 11330-032) and 100× N2 supplement (Invitrogen, cat# 17502-048), 50× B27 supplement (Invitrogen, cat#, 17504-044), 10 mM MEM NEAA (Invitrogen, cat# 35050-061), 100× Glutamax (Invitrogen, cat# 35050-061) and 55 mM B-mercaptoethoanol (Invitrogen, cat# 21985-023). The N2B27 media was prepared without bFGF (Invitrogen cat# 50037-M07E) and then supplement with fresh bFGF to a final concentration of 100 ng/mL at the time of use. On day 1 the N2B27 media was changed every other day for 7 days and every day for the remaining 2 days. On day 9 the N2B27 media was replaced with NutriStem media (Corning, cat#01-0005). This media was changed every other day, and any colony formation was observed and picked for expansion.

The following reprogramming enhancers were used:

100 µM sodium butyrate (Sigma-Aldrich, St. Louis, MI, USA),

1 mM N-acetyl- cysteine (NAC) (Sigma-Aldrich),

25 ug/mL Vitamin C (Vc) (Sigma-Aldrich),

2 μM Thiazovivin (Sigma-Aldrich).

Lentivirus-based reprogramming was used as an alternative to episomal reporgramming. Third-generation SIN Lentiviral vectors (LVs) were produced by co-transfection of HEK 293T cells using Polyethylenimine and packaging plasmids pMDL-g/pRRE, pMD2-VSVg and pRSV-Rev, as well as one of the following vectors: FUW-M2rtTA (Addgene #20342), FUW-TetO-hSOX2 (Addgene #20724), FUW-TetO-hOCT4 (Addgene #20726), FUW-Teto-hKLF4 (Addgene #20725), FUW-TetO-hMYC (Addgene #20723) or all-in-one Lenti SKOM: FUW-tetO-hSOX2, FUW-tetO-hKLF4, FUW-tetO-hOCT4 and FUW-tetO-hMYC.

All plasmids were a gift from Rudolf Jaenisch [33]. Subsequently, LVs were concentrated by ultracentrifugation of the medium of HEK 293T cells at 20,000 rpm for 2 h at 4 °C and stored at −80 °C. After optimization, ccRCC cells were sequentially transduced with lentiviral particles carrying M2rtTA and the reprogramming factors (one at a time) using multiplicity of infection = 2. Cells were let to recover and were passaged after every infection.

Isolation, expansion and maintenance of iPSCs colonies was done by picking colonies with a 27-gauge needle by carefully carving the outer boarders of the colony and cross-hatching it into smaller pieces. The colony was then pipetted using a P20 and transferred to a 24-well plate with geltrex coating and Nutristem supplemented with 10 mM of Rocki.

The cells were passaged at 75–90% confluence by removing spent media, washing the cells 2× with PBS(−/−) and adding 1 mL (1-well of a 6-well) of the accutase for 1 min. The cells were then neutralized with mTeSR and passaged onto the newly coated plates anywhere between 1:10 and 1:20 depending on the density. A half media change was performed every day. The cells were also monitored for the morphology of the cells under a light microscope. At each passage stocks of the cell lines were frozen down for later characterization. Polymerase chain reaction and agarose gel electrophoresis was conducted to check for the presence of the episome; 12–24 clones were picked from low passage iPSCs (P3-P5) for endpoint PCR. The cell pellets were digested with 100 uL of 0.05 NaOH and run for 10 min at 98 °C. Then 10 uL of 1 M Tris was added to neutralize the reaction and the DNA was further diluted by adding 100 uL of molecular grade sterile water. The primers used were oriP and EBNA-1, the backbones of the Epi5 vectors, in addition to GAPDH as a house-keeping gene. All primers were diluted to a final concentration of 5 uM. The nanodrop was used to measure the concentration of extracted DNA that was then run on a 1.5% agarose gel with 1× Sybrsafe dye and 100 bp ladder (cat#, Froggo). All the gels were visualized using UV fluorescence.

iPSC Validation

NANOG F: 5′-TGGGATTTACAGGCGTGAGCCAC-3′

NANOG R: 5′-AAGCAAAGCCTCCCAATCCCAAAC-3′

SOX2 F: 5′-CCATGCAGGTTGACACCGTTG-3′

SOX2 R: 5′-ACATGGATTCTCGGCAGACTG-3′

OCT4 F: 5′-TTGTTGGGCTGGGCTATCG-3′

OCT4 R: 5′-CCAGTTGGGAAGGGCATAGG-3′

oriP; F: 5′-TTC CAC GAG GGT AGT GAA CC-3′

oriP; R: *R*: 5′-TCG GGG GTG TTA GAG ACA AC-3′

EBNA-1; F: 5′-ATC GTC AAA GCT GCA CAC AG-3′

EBNA-1; R: 5′-CCC AGG AGT CCC AGT AGT CA-3′

Genomic DNA loading control:

GAPDH F: 5′-CGAGATCCCTCCAAAATCAA-3′

GAPDH R: 5′-GTCTTCTGGGTGGCAGTGAT-3′

### 2.4. Targeted Insertion of VHL into the Human AAVS1 Site

A previously established CRISPR/Cas9 system was employed to integrate the VHL gene into the human AAVS1 locus, located within the first intron of PPP1R12C [34]. The expression cassette, CAG-LoxP-VHL-T2A-GFP-Stop-LoxP-iRFP-pA, was synthesized by Thermo Fisher Scientific (Waltham, MA, USA) and obtained as an insert in the Gateway pENTR vector. This cassette was subsequently transferred into the AAVS1-Nst-CAG-DEST donor vector (Addgene #80489; gift from Knut Woltjen), which contains optimized AAVS1 homology arms and a CAG promoter, using the Gateway LR Clonase II Enzyme Mix (Thermo Fisher Scientific, 11791-020).

The pXAT2 plasmid, encoding Cas9 and an AAVS1-targeting single guide RNA (sgRNA), was also provided by Knut Woltjen (Addgene #80494). RCC2 and RCC3 cells were co-electroporated with the VHL donor construct and pXAT2 using the Invitrogen Neon Electroporation System under the following conditions: 500,000 cells, 1600 V, 30 ms, 1 pulse. Forty-eight hours post electroporation, cells were treated with neomycin for seven days at a concentration determined by cell line-specific kill curves. Upon emergence of epithelioid GFP-positive colonies, cells were cultured under 3% O_2_ in ATCC-PCS-400-040 medium, optimized for primary renal proximal tubule epithelial cells. Well-isolated colonies were mechanically split and expanded for genotyping and propagation.

Genomic DNA was extracted using the phenol–chloroform method. PCR amplification was performed to detect integration of the VHL construct at the AAVS1 locus, targeting regions encompassing exons 1 and 3 and adjacent genomic sequences. PCR products were sequenced via the Sanger method at the TCGA Genetic Analysis Facility. For detection of exon 1, 1 M betaine was added to the PCR reaction to enhance amplification efficiency.

The following primers were used to confirm integration at the AAVS1 site.

Dna803 forward: 5′-TCGACTTCCCCTCTTCCGATG-3′

Dna804 reverse: 5′ GAGCCTAGGGCCGGGATTCTC-3′

Dna 813 reverse: 5′-CTCAGGTTCTGGGAGAGGGTAG-3′

The following primers were used to confirm that the entire construct had been inserted into the genome.

VHL1113-F 5′-ACACGATGGGCTTCTGGTTA-3′

GFP1905-R 5′-ATGCCGTTCTTCTGCTTGTC-3′

pAAVS1-DEST-F: 5′-GCAACGTGCTGGTATTGTGCT-3′

pAAVS1-DEST-R’: 5′-CACCTGAGGAGTGAATTCTC-3′

iRFP2449 F: 5′-GCTAGCTGTGCGATCGTTTACC-3′

iRFP3200 F: 5′-TGCTTCGGAGATGAGCAAAGGA-3

Cre-ERT2 F: 5′-TGCCCCTGTTTCACTATCCAGG-3′

Cre-ERT2 R: 5′-CCAGGCTTTGTGGATTTGACCC-3′

NEO3823 F: 5′-GTGATATTGCTGAAGAGCTTG-3′

NEO3549 R: 5′-GATGCGATGTTTCGCTTGGTG-3′

Neo Probe: F: 5′-ATGGGATCGGCCATTGAACA-3′

Neo Probe: R: 5′-TCAGAAGAACTCGTCAAGAAGGC-3′

GFP Probe: F: 5′-CACCCTGACCTACGGCGTGC-3′

GFP Probe: R: 5′-GCCGTTCTTCTGCTTGTCGG-3′

VHL Exon 1 F: 5′-CGCGAAGACTACGGAGGT-3′

VHL Exon 1 R: 5′-GCTTCAGACCGTGCTATCGT-3′

VHL Exon 3 F: 5′-CAGTGTCGCTTCATCCACAT-3′

VHL Exon 3 R: 5′-CAAAAATGCCACCACCTTCT-3′

### 2.5. Flow Cytometry

Cells were counted and centrifuged at 400× *g* for 4 min, then resuspended in staining buffer (PBS(−/−) supplemented with 1% FBS). A total of 5 × 10^6^ cells were incubated with 2 µL of IgG (control) or anti-CAIX antibody in the dark for 10 min or were directly processed for quantification of GFP and iRFP expression. Following antibody incubation, cells were washed once with 1 mL of staining buffer and centrifuged at 400× *g* for 5 min at 4 °C. The pellet was resuspended in 300 µL of staining buffer containing 0.5 µL of DAPI, and samples were analyzed using a Gallios Flow Cytometer (Beckman Coulter, Brea, CA, USA).

### 2.6. Immunocytochemistry

Immunocytochemistry was performed using a modified version of a previously described protocol [35]. Briefly, cells and organoids were washed with phosphate-buffered saline (PBS(−/−) and fixed in formalin. After fixation, samples were washed three times with PBS(−/−) and incubated for 2 h in blocking buffer (PBS(−/−) supplemented with 0.1% Triton X-100 and 10% fetal bovine serum [FBS]). Primary antibodies were applied and incubated overnight at 4 °C. Following five washes with PBS(−/−) containing 0.1% Triton X-100, samples were incubated with secondary antibodies for 2 h at room temperature. After additional washes, nuclei were counterstained with DAPI, and samples were mounted in PBS(−/−) containing 50% glycerol.

The following antibodies were used: anti-CAIX (clone 303123, R&D Systems, Minneapolis, MN, USA) 1:100, anti-vimentin (Santa Cruz Biotechnology, Dallas, TX, USA, sc-6260), anti-Pax2 (Abcam, Cambridge, UK, Ab79389), anti-OCT4 (Abcam, Ab181557), anti-SOX2 (Abcam, ab92494), NANOG (Cell Signaling #4903, Danvers, MA, USA), VHL (SC-135657, Santa Cruz Biotechnology). The secondary antibodies used were as follows: anti-rabbit IgG Cy5, anti-rabbit IgG 488, anti-rabbit IgG-594, anti-mouse IgG488, anti-mouse IgG 594 (Santa Cruz Biotechnology: used at 1/100 dilution)

All images were taken on the Quorum WaveFX Spinning Disc Confocal System equipped with CSU X1 Confocal Scanner Unit (Yokogawa, Musashino, Japan), ImagEM EM-CDD Camera (Hamamatsu, Hamamatsu City, Japan) and a DMI6000 B Fully Automated Inverted Research Microscope (Leica, Wetzlar, Germany). Images were acquired and processed on the Volocity Software Version 6.3.0 (Improvision/PerkinElmer, Coventry, UK) using the DAPI (377/54 nm), FITC (475/34 nm) and Cy5 (631/28 nm) wavelengths.

### 2.7. KO of VHL in Normal ES Cells

The ObLiGaRe doxycycline inducible system was transfected with the VHL-targeting synthetic guide RNA into an established, characterized iPS cell line [36,37]. All experiments presented in this study used the previously described doxycycline-inducible Cas9 expressing hiPSC line [37]. Undifferentiated hiPSCs were maintained and transfected in the feeder-free and chemically defined culture system DEF-CS 500 (Cellartis by Takara Bio Europe, Gothenburg, Sweden). Cells were passaged every three to four days using TrypLE Select (Gibco, Waltham, MA, USA) and single cells reseeded onto fresh DEF-coated plateS using DEF-CS media with 10 µM Rock inhibitor (Tocris Bioscience, Bristol, UK). Quality control of the cells was based on morphology, karyotype and pluripotency characteristicS of the line. Doxycycline-inducible Cas9-expressing 293 T and A459 cells were cultured and transfected in DMEM-glutamin (Gibco) supplemented with 10% fetal bovine serum (Gibco) and 1% penicillin/streptomycin (Gibco). All cells were maintained at 37 °C in a humidified 5% CO_2_ incubator.

### 2.8. Kidney Organoid Differentiation

CRISPR-targeted and non-mutant isogenic control hiPSCs were differentiated into kidney organoids using a modified version of the protocols published by Takasato et al. [35,38] and Morizane et al. [39] involving inhibition of glycogen synthase kinase-3B (GSK3β) and stimulation by FGF9. The day before differentiation, hiPSCs were dissociated into single cells from 100% confluent cultures with TrypLE Select (Gibco) and plated at 15,000 cells/cm^2^ in a 6-well plate pre-coated with Geltrex Reduced Growth Factor Basement Membrane Matrix (Gibco) in DEF-CS 500 medium with 10 µM Rock inhibitor. Cells were incubated overnight at 37 °C and 5% CO_2_. On the next day cells were rinsed with PBS(−/−) and treated with 6 µM CHIR99021 (Tocris Biotechne, Bristol, UK) in APEL2 basal medium (STEMCELL Technology, Vancouver, BC, Canada) supplemented with 5% Protein Free Hybridoma Media (PFHM-II; Gibco) and 1% penicillin/streptomycin (Thermo Fisher Scientific) for 4 days, followed by 5 days with FGF9 (200 ng/mL), during which the medium was changed every second day. At day 10, cells were dissociated into single cells using TrypLE select, plated at 1.2  ×  10^6^ cells per well in AggreWell400 (Stemcell Technologies, Vancouver, BC, Canada) and treated with 3 µM CHIR99021, 200 ng/mL FGF9 and 10 µM Rock inhibitor in APEL2 basal medium. Plates were centrifuged at 200× *g* for 15 s at room temperature and incubated at 37 °C and 5% CO_2_ for 2 days. After 2 days (day 12), organoids were transferred to ultra-low adhesion plates in a shaker incubator (at 37 °C and 5% CO_2_) rotating at 100 rpm. On days 12 and 14, organoids were treated with 200 ng/mL FGF9 in APEL2 basal medium. From day 14 onwards, suspension culture continued in factor-free APEL2 basal medium for up to 17 days, with medium change three times per week

### 2.9. CRISPR-Cas9 Design and Generation

Paired-sgRNAs targeting specific genes were designed. Plasmid guide RNA was generated by cloning targeting oligos into a U6 promoter-driven backbone vector using digestion ligation cloning. Chemically synthesized oligoribonucleotides were manufactured by Synthego.

VHL-sg56 C*U*C*UUCCGGGCCGGACUCCU synthego modified EZ Scaffold VHL-sg57 C*G*C*GCGUCGUGCUGCCCGUA synthego modified EZ Scaffold.

#### PCR Analysis

Genomic DNA was isolated either using Puregene cell and tissue kit (Qiagen, Venlo, The Netherlands) or QuickExtract DNA Extraction Solution (Lucigen, Epicenter, Middleton, WI, USA) according to the manufac-turer’s recommended protocols. Gene specific primers were designed using Primer-BLAST tool. V2.11.0 [40].
**Primer****Target Gene****Sequence**Location *171-FVHLGCGTTCCATCCTCTACCGAG5084–5103172-RVHLGCTTCAGACCGTGCTATCGT5589–5608173-FVHLCGTTACAACGGCCTACGGT5010–5028174-RVHLTTCAGACCGTGCTATCGTCC5587–5606175-FVHLCTGGATCGCGGAGGGAATG5198–5216176-RVHLGGCTTCAGACCGTGCTATCG5590–5609* Homo sapiens von Hippel-Lindau tumor suppressor (VHL), RefSeqGene (LRG_322) on chromosome 3.

Amplifications were performed using 100 ng of genomic DNAs extracted with PureGene kit. PCR reactions contained 0.25 µM final concentration of each forward and reverse primers. The following thermal cycling condition was used: 1 cycle initial denaturation (2 min at 98 °C), 35 amplification cycles (10 s at 98 °C, 10 s at 60 °C, 5 s at 72 °C) and 1 cycle final extension (5 min at 72 °C).

## 3. Results

### 3.1. Healthy Renal Tissue Adjacent to the ccRCC Tumor Reprogram with High Efficiency

The lines for adjacent normal (AN) tissue, AN2 and AN3, were analyzed for PAX2 and VIMENTIN to verify their renal epithelial origin (Supplementary Figure S1A). Primary cell cultures from the cancerous ccRCC tumor cells, RCC2 and RCC3, were derived based on the expression of carbonic anhydrase IX (CAIX), a downstream target of HIF1α and a clinically relevant marker of hypoxia [26]. ccRCC cells, due to the loss of VHL function, constitutively express CAIX under hypoxic (3% O_2_) and normoxic (21% O_2_) conditions [41], while wild-type cells only express CAIX under hypoxic conditions (Supplementary Figure S1B). Sanger sequencing demonstrated that the AN2 cells are heterozygous for 481 C>T nonsense mutation in exon 3 of the VHL gene, and the tumor counterpart RCC2 is hemizygous. AN3 is wild type for VHL, and RCC3 is hemizygous for the 337 C>T nonsense mutation in exon 1 of the VHL gene (Supplementary Figure S1C). There were also observable mutations in other genes associated with ccRCC (see list in Supplementary Figure S1D).

Before reprogramming adjacent normal cells and ccRCC patient cell lines, we determined if normal renal tissue could be reprogrammed. In previous studies, we have successfully used the Epi5 episomal vector system to reprogram renal epithelial cells in pediatric urine samples [42]. Here we used healthy renal epithelial cells isolated from adult urine as described previously [43] as a control. We were able to obtain identifiable iPSC colonies that expressed the pluripotency proteins OCT4, SOX2 and NANOG by day 21 with 0.02% efficiency (Supplementary Figure S2A). Using the same conditions, adjacent normal tissues, AN2 and AN3 reprogrammed with 0.05% and 0.06% efficiency. The obtained iPS cell lines stained positive for the endogenously pluripotency markers OCT4, SOX2 and NANOG and could be passaged indicating a stable pluripotent state (Supplementary Figure S2B).

### 3.2. ccRCC Cell Lines Are Resistant to a Variety of Reprogramming Strategies

Reprogramming of the ccRCC tumor lines using the same Epi5 system was not successful even after a long culture period of 35–40 days. Some small colonies did appear but did not grow. These remained negative for the NANOG pluripotency protein and did not survive passaging. To better understand the potential barriers to reprogramming ccRCC cells, we systematically assessed several parameters that were reported to enhance reprogramming. Cancer cell line RCC2 showed poor transfection efficiency with a variety of liposomal transfection agents, so electroporation was tried. After extensive optimization, 20–25% electroporation efficiency was achieved. Despite the initial proliferation burst and signs of early epithelialization, compact cell colonies only emerged more than forty days after electroporation and were negative for the SSEA3 pluripotency marker. We also tried to boost reprogramming factor expression by a second round of electroporation of the Epi5 vectors on day 14 or day 21 post first electroporation. This did not improve reprogramming.

Since the relatively low electroporation efficiency of ccRCC cell lines could limit the use of the episomal vector system, we proceeded to deliver the reprogramming factors using lentiviral particles. RCC2 demonstrated 77% transduction efficiency after optimizing the multiplicity of infection (Supplementary Figure S3). RCC2 was sequentially infected with lentiviral particles carrying the constitutively active reverse tetracycline transactivator (FUW-M2rtTA) and the doxycycline-inducible reprogramming factors as four separate vectors or as a single construct (Lenti OKOM: FUW-tetO-hSOX2, FUW-tetO-hKLF4, FUW-tetO-hOCT4 and FUW-tetO-hMYC).

**Figure 1:**
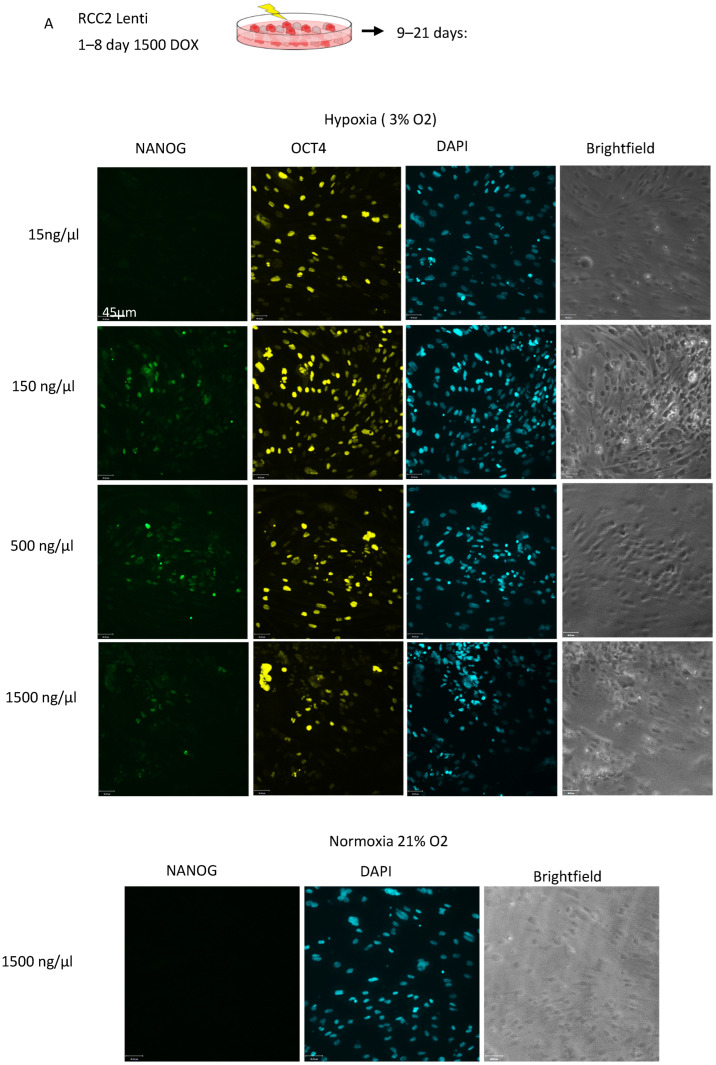

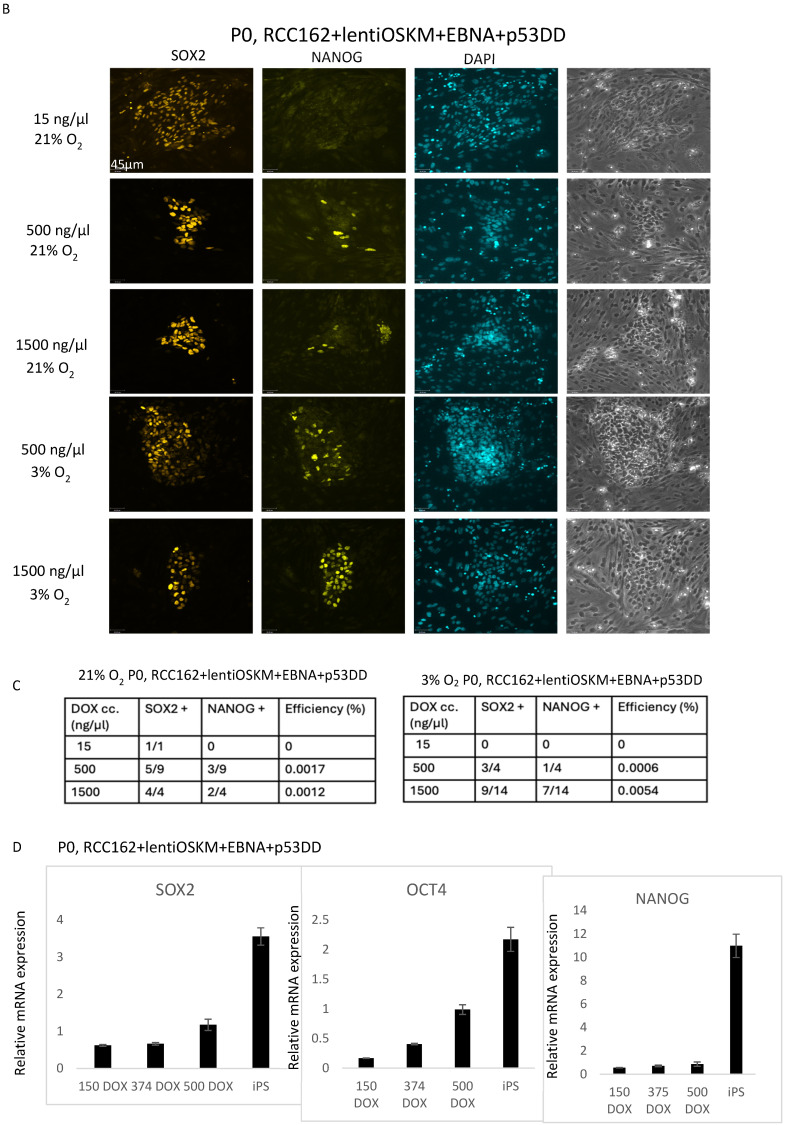
ccRCC cells show low rate of pre-iPS colony formation and are dependent on the continuous presence of extrinsic reprogramming factors. (**A**) RCC2 cells were infected with lentiviral particles carrying the SKOM reprogramming factors and the M2rtTA reverse tetracycline transactivator (RCC2 + lentiSKOM). Reprogramming was initiated by 1500 ng/mL DOX treatment for 8 days. The RCC2 + lentiSKOM cells were then electroporated with the Episomal vectors carrying EBNA1 (EBNA) and a dominant negative mutant of p53 (p53DD) to enhance lentivirus-based reprogramming prior to inducing reprogramming by DOX treatment. To optimize the expression level of the LV reprogramming factors, DOX was used in different concentration (ranging from 1500 ng/mL to 15 ng/mL) for d9–21 of the reprogramming protocol. [mag bar = 45 µm, first panel]. (**B**) Reprograming was performed under ambient oxygen (21% O_2_) and hypoxic (3% O_2_) conditions. Formation of initial pre-iPSC colonies was evaluated on day 21 by staining for SOX2 and NANOG pluripotency markers, matching DAPI and the brightfield panel next to the IF panel. (**C**) Presence of pre-iPS colonies was assessed on day 21 by staining for SOX2 and NANOG pluripotency markers. Reprogramming efficiency is expressed as the number of compact colonies on day 21 over the number of plated cells. (**D**) mRNA expression of pluripotency markers was evaluated 21 days after inducing reprogramming with DOX treatment. Human iPSCs were used as a positive control [50]. Control RCC2 cells did not express SOX2, OCT4 and NANOG.

The stem cell state is better maintained in hypoxic conditions, but a burst of oxidative phosphorylation is required to initiate reprogramming [44,45,46]. Therefore, we tested reprogramming of RCC2 in ambient oxygen and hypoxia conditions. Furthermore, lowering the DOX concentrations after the initial 8 days has previously been shown by us to be beneficial for reprogramming [47] (Figure 1A). Reprogramming was induced in the RCC2 + lentiSKOM cells by treatment with varying levels of doxycycline (DOX) for varying lengths of time at 3% O_2_ and 21% O_2_. No iPSC colonies were produced as assayed for NANOG expression using 1500 ng/mL Dox for 21 days at 21% O_2_ (Figure 1B). Reprogramming experiments were repeated under hypoxic conditions (3% O_2_) to determine if hypoxia could enhance reprogramming, and after day 8 the DOX concentration was lowered. Using 3% oxygen and 15 ng/mL DOX, no iPSC pre-colonies were observed. Increased DOX concentrations correlated with increased expression of SOX2 and OCT4 at mRNA and protein levels. A few NANOG+ cells were observed when DOX concentration was continuously high (150–1500 ng/mL) and the reprogramming was performed under hypoxic conditions (3% O_2_). This observation indicates that RCC2-iPSC colonies remained dependent on exogenous OSKM expression, and the endogenous reprogramming network was not activated.

The lentivirus reprogramming vector contained the four Yamanaka factors (Sox2, Klf4, Oct3/4, c-Myc), while the Epi5 system also contained Lin28, mp53DD9 (p53 carboxy-terminal dominant-negative fragment, a p53 suppressor) and EBNA1 (episome replication promoter protein). The dominant negative mutant mp53DD reportedly enhances reprogramming efficiency [48]. To assess whether the extra genes could increase iPSC colony formation and NANOG positivity, ccRCC cells were transduced with lentiviral particles carrying the SKOM reprogramming factors and additionally electroporated with pCE-mp53DD and pCXB-EBNA1, resulting in RCC2 + LV-SKOM + EBNA 1 + mp53DD cells. DOX was applied at 1500 ng/mL for days 1–8 and then 15–1500 ng/mL DOX for 9–21 days to induce the lentiviral vectors. Reprogramming was also carried out in hypoxia and ambient oxygen conditions. These additional episomal factors did not boost iPSC colony forming efficiency. Initial colonies were observed at day 21 that were Sox2 positive but had few NANOG positive cells except for the 1500 ng/mL DOX concentration for 21 days (Figure 1C). However, none of the colonies could be sustained in culture (Figure 1D). The Sox2 and Oct4 expression was likely entirely supported by the exogenous factors and never reached threshold levels required for independent growth. Additionally, we also tried to supplement reprogramming media with the reprogramming enhancer, histone deacetylase inhibitor sodium butyrate, however, experimental outcomes remained the same. Overall, high DOX concentration and hypoxic conditions were necessary for the iPSC colony formation of ccRCC cells, but DOX-independent iPSC lines could not be established.

### 3.3. Expression of Wild-Type VHL Gene in ccRCC Cells Through Knock-In Gene Engineering Partially Corrects the Hypoxia Response, Resulting in an Increased Reprogramming Rate

An initial burst of oxidative phosphorylation (OXPHOS)-driven respiration, and thus a normoxic environment, is critical for initiating cellular reprogramming. However, ccRCC cells remain pseudo-hypoxic with low OXPHOS capacity even under standard atmospheric conditions (21% O_2_), as evidenced by constitutive CAIX expression. We therefore hypothesized that restoring VHL function in ccRCC cells could re-establish a normoxic state conducive to reprogramming. RCC2 cells exhibit loss of heterozygosity at the VHL locus, expressing only the mutant allele. In contrast, AN2 cell lines are derived from tissue with normal histology, retain one wild-type VHL allele and undergo reprogramming readily, consistent with our hypothesis that introducing a single wild-type VHL allele into RCC2 cells should similarly enable reprogramming. Our approach was to knock in a floxed wild-type VHL allele using a conditional construct that can be removed after reprogramming has occurred. By first restoring VHL function, we aimed to re-establish normoxia and thereby enable reprogramming. Once reprogramming was complete, the introduced wild-type allele would be removed by expressing Cre recombinase, to revert the cells to their cancerous genotype. We hypothesized that this strategy would allow us to capture and manipulate the earliest events in ccRCC initiation within a developmental context.

To correct the VHL gene in the RCC2 cancerous line, the CAG promoter-loxP-VHL-T2A-GFP-STOP-loxP-iRFP construct was inserted into the AAVS1 locus (VHL WT GFP/-). AAVS1 is a safe-harbor locus within the PPP1R12C gene that allows potent expression of transgenes [34] (Supplementary Figure S4). The T2A self-cleaving peptide assured that pVHL and GFP were expressed from the same mRNA as separate proteins. The transgenic loxP-WT VHL-T2A-GFP-STOP-loxP-iRFP cassette had two advantages. First, the conditionally active form of Cre DNA recombinase, ERT2-Cre-ERT2, enabled the regulated removal of the wild-type VHL copy in the presence of 4-hydroxitamoxifen, restoring the genetic background of the ccRCC specimen. Secondly, excision of the VHL-T2A-GFP cassette permitted the expression of the iFRP color; this allowed the monitoring of the VHL status by FACS. No color expression indicated the cells did not receive the vector and remain unchanged (Supplementary Figure S4A–C). Emerging RCC2 + VHL WT GFP+ were composed of compact, small epithelioid cells, resembling the normal renal epithelial cells (Supplementary Figure S4D,E). The GFP negative RCC cells remained large with loose association to each other, often presenting a spindled shape, characteristic for ccRCC cells (Supplementary Figure S4F,G). Within the mixed population of RCC2 + VHL WT GFP+ and GFP- cells, under normoxic condition (21% O_2_), the RCC2 + VHL WT GFP+ cells had decreased membranous expression of the hypoxia indicator CAIX, while the RCC2 + VHL WT GFP- (VHL removed) cells retained strong expression of CAIX. As a positive control, CAG-loxP-T2A-GFP-STOP-loxP-iRFP was transiently co-expressed in HEK293 cells with ERT2-Cre-ERT2. Cre activation efficiently replaced GFP with iRFP, verifying the integrity of the expression cassettes (Supplementary Figure S4H–J).

**Figure 2:**
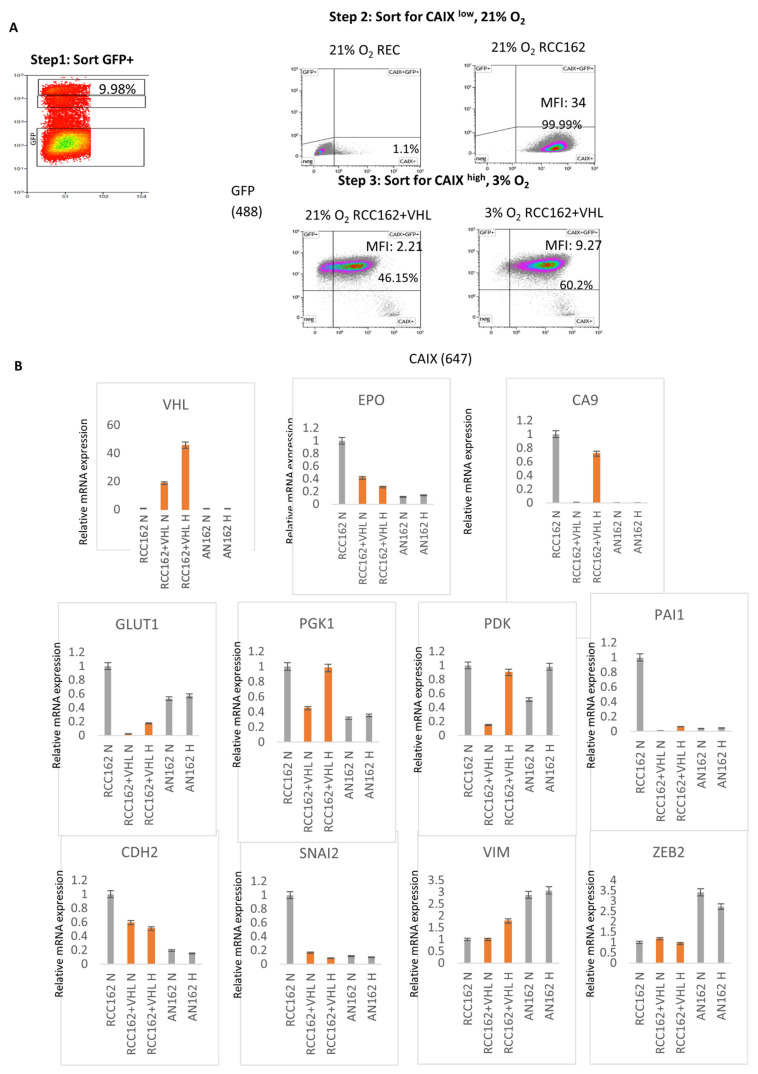
Re-expressed VHL inhibits hypoxia pathway. (**A**) The panel summarizes the strategy to select for electroporated ccRCC cells with improved normoxia/hypoxia response. ccRCC cells expressing the VHL-T2A-GFP construct were enriched by sorting for GFP+ cells with low CAIX expression under normoxic conditions (Steps 1 and 2). Subsequently, the cells were kept under hypoxic conditions and were sorted for high CAIX expression to ensure their ability to respond to hypoxia (Step 3). As expected, healthy renal epithelial cells (REC) did not express the CAIX hypoxia marker under ambient oxygen, while over 99% of RCC2 cells expressed CAIX even under ambient O_2_ levels with an MFI of 34. In comparison, the VHL-T2A-GFP expressing RCC2 cell population (RCC2 WT VHL GFP+) partially recapitulated its response to oxygen levels. Mean fluorescence intensity (MFI) at 21% O_2_ level was less than 25% of the hypoxic score (2.21 vs. 9.27). (**B**) Upper panel: VHL expression and hypoxia pathway activity was assessed by qRT-PCR in normoxic RCC2 cells, and in RCC2 WT VHL GFP+ and AN2 cells under 21% and 3% O_2_ levels. N: normoxia; H: hypoxia. Middle panels: Glycolysis is upregulated by the hypoxia induced factors. Restoration of VHL expression partially normalized the ccRCC cells’ pseudohypoxia condition and lowered the expression of the downstream glycolysis- and epithelial-to-mesenchymal transition related genes (lower panel).

To enrich for oxygen responsiveness in VHL-corrected RCC2 cells and preserve the tumor cell heterogeneity, we developed a three-step strategy. RCC2 + VHL WT GFP+ cells were sorted for GFP and cultured in renal epithelial cell (REC) media under hypoxic conditions. Second, RCC2 + VHL WT GFP+ cells were cultured at 21% O_2_ and normoxia-responding cells were selected by sorting for the CAIX^low^ population. Finally, we confirmed that the final RCC2 + VHL WT GFP+ population could react to hypoxia by culturing cells in a 3% O_2_ chamber and sorting for the CAIX^high^ population (Figure 2A). After further propagation, the final RCC2 + VHL WT GFP+ pool showed improved oxygen level response. Under normoxic conditions, about 46% of the cells still showed CAIX positivity; however, the median fluorescent intensity was about four-fold lower than the unmanipulated RCC2 cell line, indicating improved oxygen response (MFI of 2.21 Vs. MFI of 9.27) after step 3 (Figure 2A).

As an initial assessment of VHL function, mRNA expression of established hypoxia markers (EPO, CAIX, PAI1) [49], glycolysis enzymes (GLUT1, PGK1, PDK) and epithelial-to-mesenchymal transition (EMT) markers (CDH2, SNAI2, VIM, ZEB2) were evaluated in the RCC2 + VHL WT GFP+ cells (Figure 2B). Importantly, the hypoxia marker CAIX showed decreased mRNA level under 21% O_2_ condition versus 3% O_2_. The expression of glycolytic enzymes significantly decreased upon VHL re-expression, with a notable sensitivity to oxygen levels. EMT marker expression, however, did not show a statistical difference between VHL-expressing and non-expressing ccRCC cell lines or when compared to the adjacent normal renal epithelial cells. Based on the morphological and gene expression characteristics, we concluded that ectopic VHL expression has partially restored the renal epithelial traits in patient-derived ccRCC cell lines.

**Figure 3:**
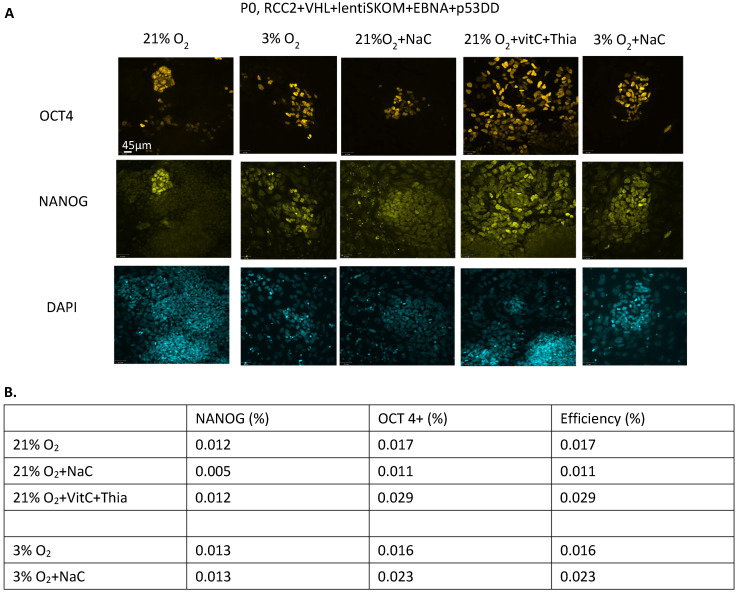
VHL re-expression in ccRCC cells results in increased reprogramming capacity. (**A**) VHL re-expressing RCC2 cells were reprogrammed by electroporating EBNA and p53DD and infecting with lentiviral particles carrying the SKOM reprograming factors. Reprogramming was performed under ambient oxygen (21% O_2_) and hypoxia (3% O_2_) conditions, and media was supplemented with N-acyl-cysteine (NaC), Thiazovivin (T) or ascorbic acid (vC), as indicated. NANOG and OCT4 positivity was assessed by immunocytochemistry. Representative immunocytochemistry images shown. [mag bar = 45 µm, first panel]. (**B**) Efficiency of colony formation was assessed by calculating the number of colonies per number of cells transfected.

To determine if the VHL-corrected cells had improved reprogramming, they were transduced with lentiviral particles carrying FUW-M2rtTA and the human OSKM genes expressed from a single vector (RCC2 + VHL WT GFP+ with LentiOSKM). Additionally, EBNA-1 and p53DD were electroporated into the RCC2 + VHL WT GFP+ with LentiSKOM cells right before the initiation of reprogramming. This resulted in OCT4 and NANOG double positive iPSC colonies (Figure 3A: Column 1,2). The time to iPSC colony formation was notably shortened in the VHL-corrected ccRCC cells from 43 days to 24 days.

The medium was supplemented with oxidative stress inhibitors N-acetyl-cysteine, or a combination of ascorbic acid and Thiazovivin, that were appplied between the 8–24 days of the protocol, which helped to improve reprogramming efficiency (Figure 3A Column 3, 4, 5 and Figure 3B). Despite the improvement in initial reprogramming efficiency, sustainable colonies were not formed.

### 3.4. Knockout of VHL in Normal iPSC Reduces Cell Survival

To confirm that VHL also has a role in iPSC colony stability, we used the Cas9/CRISPR system to knock out the WT VHL gene in iPS cells (Supplementary Figure S5A). There are three VHL gene splice variants; therefore, we targeted the first exon. The ObLiGaRe doxycycline-inducible system was transfected with the VHL-targeting synthetic guide RNA into an established, characterized iPS cell line [36,37]. Three replicates were produced and not clonally selected. Therefore each cell pool consisted of wild-type, heterozygous knock out and homozygous knock out cells (Supplementary Figure S5B).

**Figure 4:**
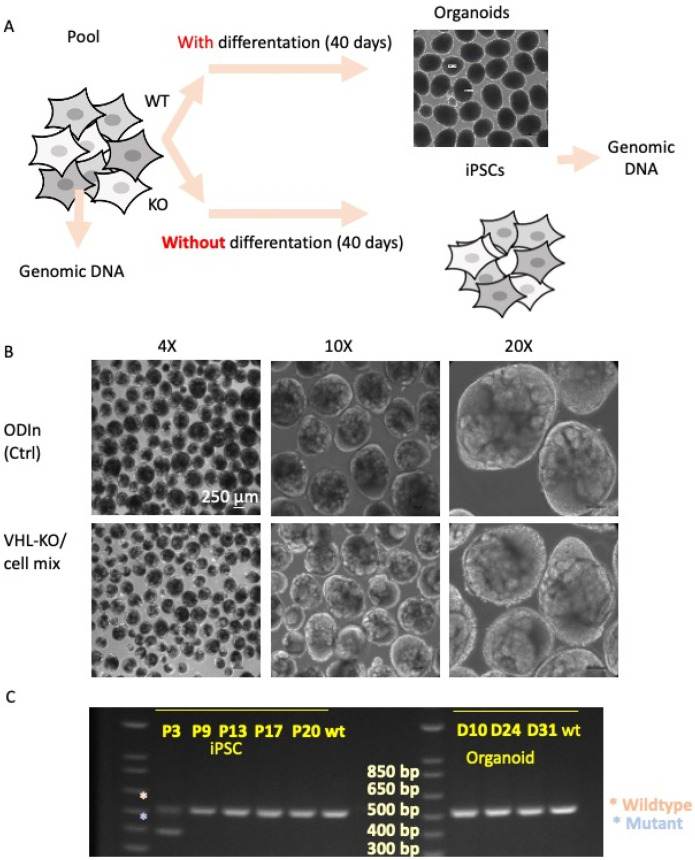
iPSCs were edited using CRISPR-Cas9 to remove the VHL gene. The pool of cells contained unedited (VHL+/VHL+), heterozygous (VHL-/VHL+) and homozygous null (VHL-/VHL-) cells. (**A**) The pool of cells were grown as kidney organoids for 40 days to allow for differentiation, and as controls the cells were maintained in typical iPSC maintenance conditions for 40 days. [mag bar = 250 µm, first panel] (**B**) iPSC cultures and organoids were compared to unedited cells. The organoids from the edited pool looked identical to the organoids from the unedited cells. (**C**) PCR revealed that during iPSC culture the edited cells are present at P3 but are not detected at P9-P20. For the organoids, by day 10 there are no edited cells present.

Pools of iPSC VHL-KO cells were grown either as iPSC monolayers or as kidney organoids for 40 days (Figure 4A). Interestingly, when the iPSC VHL-KO pool was assayed for VHL status at different passages, VHL-KO iPSCs were detected at passage 3 (P3) but were no longer present by passage 9 (P9). Organoids were also examined for VHL-KO cells. There were no morphological differences between the VHL-KO iPSC pool organoids and organoids generated exclusively from wild-type cells (Figure 4B). However, closer inspection of organoid composition revealed that organoids derived from the VHL-KO iPSC pool contained no detectable VHL-KO cells. Similarly, organoids generated from early-passage (P1–P3) VHL-KO iPSC pools lacked KO cells as early as day 10 of the differentiation process (Figure 4C). We concluded that the VHL gene is required for the stability and survival of pluripotent cells due to the lack of edited VHL-KO cells. This data identified a bottleneck in the reprogramming of ccRCC. It may be possible to initiate reprogramming, but the lack of the VHL gene means stable lines are unlikely to be produced.

## 4. Discussion

Cancer organoid cultures are emerging as exciting systems that complement the current conventionally controlled 2D in vitro models with the added complexity of 3D, multi-tissue structures. Samples of patient-derived cells from established malignancies are valuable for drug screening, determining growth factor dependency and modeling tumor heterogeneity. It is, however, unclear whether the cancer progenitor cells present in the organoids will recapitulate early oncogenic events or if a more primitive cancer progenitor state exists that could only be captured by driving cells through a full differentiation program, starting from the pluripotent state [19,51]. This approach was applied to solid tumors by Kim et al. 2013 [18], where pancreatic intraepithelial neoplasia precursor lesions were generated and shown to undergo a stepwise differentiation to invasive pancreatic ductal adenocarcinoma. Similarly, the Li–Fraumeni syndrome-related osteosarcoma precursor has been modeled by differentiating patient-derived iPSCs into osteoblast lineage. Despite its potential, cancer cell reprogramming is only sparingly used as a premalignancy model due to the difficulties in obtaining patient-derived cancer-iPSC lines and their targeted differentiation [21]. We experienced this in this study when we attempted to reprogram ccRCC cells. Although healthy renal cells and the ccRCC patient-derived adjacent normal renal cells reprogrammed successfully, the tumor cells did not despite using a variety of different reprogramming methods. Our present work aimed to address these shortcomings by the re-expression of the most affected tumor suppressor gene in ccRCC, VHL. It is the first step toward our overarching goal to develop a 3D organoid-based in vitro system that recapitulates premalignant stages of ccRCC and enables their molecular characterization while preserving heterogeneity.

The function of VHL during reprogramming is obscure. In ccRCC cells, during cellular respiration, HIF’s initiate glycolysis and in parallel downregulate mitochondrial oxidative phosphorylation (OXPHOS) through the transcriptional downregulation of PGC1α, resulting in the characteristic energy map of ccRCC (Warburg effect) [52,53,54,55]. Although stem cells, similar to cancer cells, favor the glycolytic pathway [56], it has been demonstrated that during the initial stages of reprogramming there is a mandatory burst of OXPHOS prior to the switch to the glycolytic pathway [29,57]. The accumulation of HIFs, mediated by VHL loss, may prevent this OXPHOS burst and interfere with somatic cell reprogramming in ccRCC cells. Restoring the OXPHOS burst might be an essential factor in restoring reprogramming ability. Secondarily, mesenchymal–epithelial transition (MET) is required for cellular reprogramming [47], but VHL mutations reduce MET and enhance Epithelial–Mesenchymal transition (EMT) [58].

Interestingly, the loss of VHL inhibits the proliferation of embryonic renal progenitor cells. Cargill et al. [59] reported that VHL loss in embryos induces a metabolic shift toward glycolysis resulting in reduced proliferation and decreased glomeruli. Similarly, Mack et al. [60] demonstrated that VHL knockout in mouse ES cells reduced cell proliferation. Our findings are consistent with these observations. We showed that the removal of VHL from wild-type human iPSCs leads to significant cell loss, likely due to severely impaired proliferation. Therefore, although the loss of functional VHL appears to enhance ccRCC proliferation and survival, its loss is detrimental during the transition to iPSCs, as evidenced by the inability of ccRCC cells to generate iPSCs.

Taken together, the reduction in the OXPHOS burst, the reduction in MET and the reduction in proliferation of iPSCs due to the loss of functional VHL would indicate that the re-expression of VHL should restore the renal epithelial cell properties and the OXPHOS respiration burst required for the initiation of reprogramming and the stabilization of iPSC lines. Previous studies have demonstrated that the overexpression of the wild-type VHL can reset the hypoxia response and gene expression networks in pluripotent stem cell lines and the established ccRCC model cell line 786-0 [61,62,63].

We developed a multi-step cell sorting protocol to select for a ccRCC + VHL cell population that regained hypoxic responsiveness. After an initial sorting for RCC/VHL WT GFP+ cells, our method used a functional readout of VHL, hypoxia-induced carbonic anhydrase IX (CAIX) protein expression. Additionally, when switching from hypoxic to normoxic culture conditions, RCC + VHL WT GFP+ cells were further selected for their ability to downregulate CAIX. Unlike single-cell derived colonies, this multi-step selection maximized the diversity of patient-derived ccRCC cells that demonstrated restored VHL HIF regulation. Similar to the 786-0 tumor lines, we observed an increased rate of OXPHOS, and the metabolic gene expression shifts due to the re-expressed WT VHL. Concomitantly, hypoxia-related protein expression, a hallmark of ccRCC, decreased when functional VHL expression was re-established, and key enzymes of glycolysis, downstream of hypoxia, showed reduced expression. All this indicates that the reintroduction of a functional VHL restored normal metabolic function to the cells. This was also observed in a study by Krieg [62].

As expected, the re-expression of VHL increased the rate OCT4+ and NANOG+ iPSC colonies generated from reprogrammed ccRCC cells, but unexpectedly, these initial iPSC colonies we not able to form stable colonies. The expression of the pluripotency protein NANOG in the VHL-corrected ccRCC line indicates the initial colonies we achieved are in an intermediate reprogramming state [64], and despite the presence of a WT VHL gene, the cells are retaining some of their tumor cell properties and cannot fully transition to a pluripotent state due to other mutated genes that contribute to ccRCC.

Additionally, ccRCC cells are highly sensitive to oxidative stress [65]. We tested N-acetylcysteine (NaC), ascorbic acid (vitC) and rock inhibitor, agents known to reduce oxidative stress or enhance reprogramming [66,67,68,69], but none promote iPSC colony formation as measured by NANOG expression

The lack of fully reprogrammed cells from ccRRCC VHL+-restored patient tumor cells could be related to the other gene mutations that are also present in ccRCC lines. Considering these results, it is possible that additional mutations present in the cancerous cells interfere with reprograming. The most common non-VHL mutations in ccRCC can be grouped as chromatin modifiers located in the proximity of the VHL gene (BAP1, PBRM1, SETD2) or distally- (KDM6A, KDM5C, ARID1A, SMARCA4) and PI3K/AKT-related signaling modifiers. Correct chromatin modification is critical for stabilizing iPSC colonies [47,70]. Interestingly, KDM5C and KDM6A demethylate at the H3K4 and H4K27 positions, influencing pluripotency [47]. Additionally, KDM6A directly senses oxygen tension as O_2_ is its cofactor, and BAP1 is an activator of KDM6A and the H3K4 methylation-based open chromatin [71]. Additionally, PBRM1, the recognition subunit of the SWI/SNF complex, recognizes and binds to the nucleosomes via direct interaction with H3K4me3. PBRM1 bromodomains variably influence nucleosome interactions and cellular function [72,73]. Future studies should compare reprogramming rates across ccRCC tumors with different secondary mutations affecting histone methylation status, nucleosome remodeling complexes and ccRCC-specific signaling pathways (PI3K/AKT-mTOR). A systemic comparison may identify ccRCC molecular subcategories compatible with reprogramming or advise on their optimization of ccRCC cells.

## 5. Conclusions

VHL loss has detrimental consequences on clear cell renal carcinoma cell reprogramming. We demonstrate that the VHL gene affects reprogramming initiation and the survival of reprogrammed cells. The restoration of the VHL gene was sufficient to increase the rate of reprogramming initiation and NANOG expression, but it did not allow for full reprogramming of the ccRCC cells to form stable, proliferating iPSC colonies. Other mutated genes associated with ccRRCC may be involved in the stabilization of iPSC lines. A systematic approach to correct these genes may lead to successful iPSC line establishment from ccRCC patient samples.

## Data Availability

All data is contained within the manuscript and Supplementary data section.

## Supplementary Materials

The following supporting information can be downloaded at https://www.mdpi.com/article/10.3390/cancers17223693/s1, Figure S1: Characterization of the patient-derived renal epithelial and clear cell renal cell carcinoma cell lines; Figure S2: iPSC can be obtained from the Adjacent Normal renal tissue from patient AN2 and AN3; Figure S3: Successful Lentivirus delivery of reprogramming factors to RCC2 patient cells; Figure S4: VHL re-expression and reprogramming strategy; Figure S5: CRISPR design for VHL knock out and validation of edited cells.

## Abbreviations

The following abbreviations are used in this manuscript:

ccRCC	clear cell renal cell carcinoma
EMT	Epithelial–Mesenchymal transition
iPSC	induced pluripotent stem cells
MET	mesenchymal–epithelial transition
OXPHOS	oxidative phosphorylation
VHL	von Hippel-Lindau factor
