# Supplementary material for: VHL Gene Restoration Supports RCC Reprogramming to iPSCs but Does Not Ensure Line Stability
Source: Cancers (Basel). 2025 Nov 18;17(22):3693. doi: 10.3390/cancers17223693 (PMC12650717; doi:10.3390/cancers17223693)
Supplement: Supplementary file 1 [file cancers-17-03693-s001.zip › cancers-3822732-supplementary.pdf]

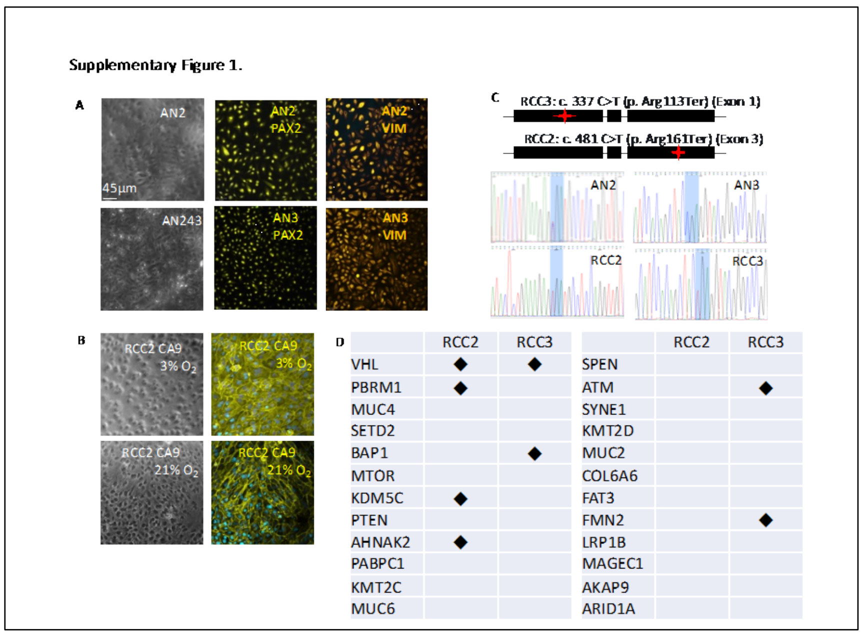

	RCC2	RCC3		RCC2	RCC3
VHL	◆	◆	SPEN		
PBRM1	◆		ATM		◆
MUC4			SYNE1		
SETD2			KMT2D		
BAP1		◆	MUC2		
MTOR			COL6A6		
KDM5C	◆		FAT3		
PTEN			FMN2		◆
AHNAK2	◆		LRP1B		
PABPC1			MAGEC1		
KMT2C			AKAP9		
MUC6			ARID1A		

Figure S1. Characterization of the patient-derived renal epithelial and clear cell renal cell carcinoma cell lines. (A) A VHL and a sporadic ccRCC patient's nephrectomy specimens were used to establish cell lines from clear cell renal cell carcinoma (RCC2 and RCC3) and the adjacent normal renal cortex (AN2 and AN3). Early passage AN cell lines showed nuclear PAX2 expression, confirming their renal origin. Cells showed vimentin expression, in line with their epithelial features. (B) ccRCC cell line RCC2 showed a prominent, membranous expression of the CAIX hypoxia marker. Due to the loss of VHL function, ccRCC cells express CAIX under ambient oxygen (21% O2) and hypoxic (3% O2) culture conditions. (C) AN2 and RCC2 are heterozygous or hemizygous (respectively) for the 481 C>T nonsense mutation in exon 3 of the VHL gene. AN3 is normal for both VHL genes, while the cancer cell line RCC3 is hemizygous for the 337 C>T nonsense mutation in exon 1. (D) Common ccRCC mutations (♦) were identified using Sanger sequencing for the RCC2 and RCC3 cell lines.

Supplementary Figure 2.

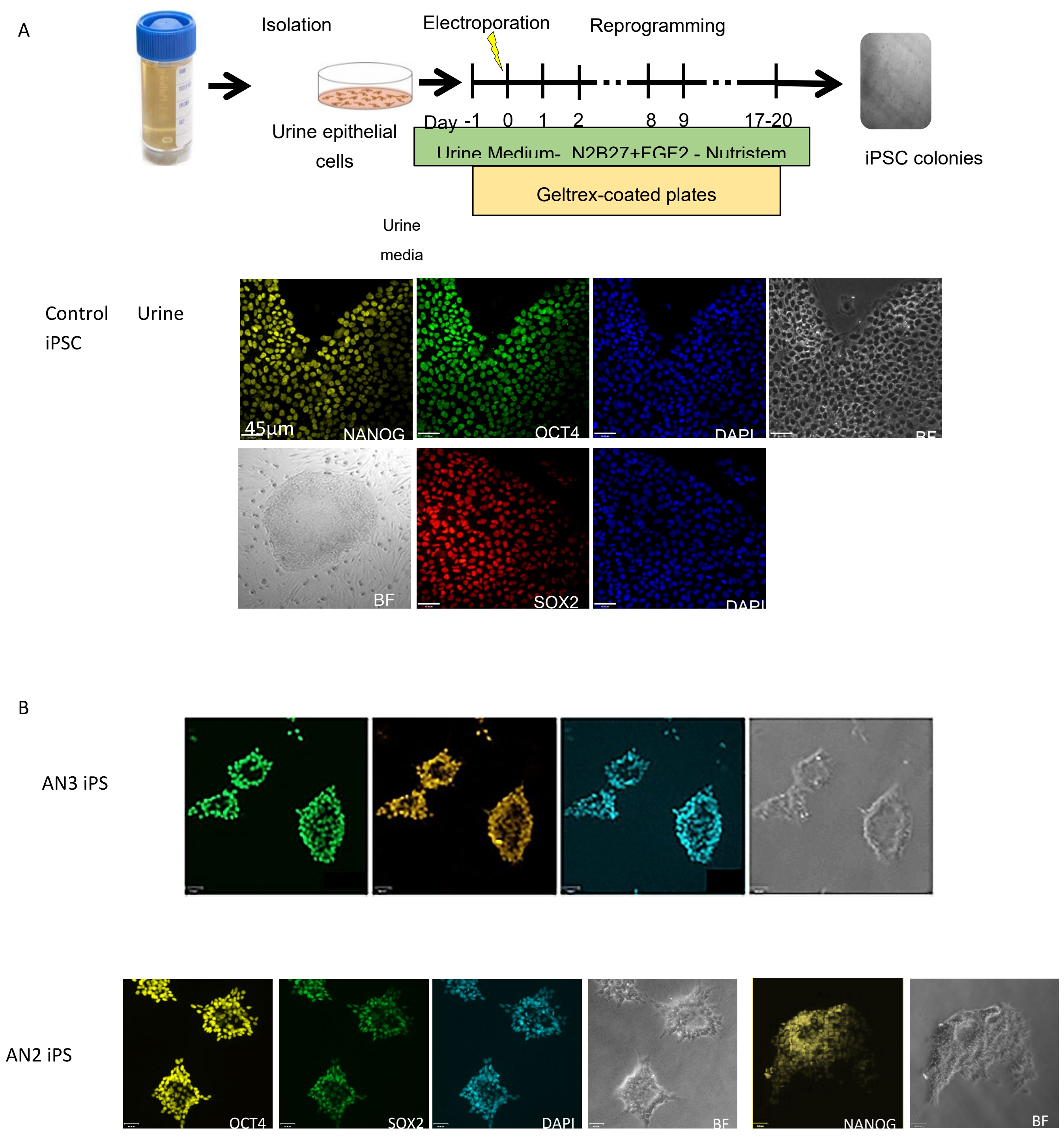

Figure S2. iPSC can be obtained from the Adjacent Normal renal tissue from patient AN2 and AN3. (A) Cells were first isolated from the urine of a healthy donor to test the reprogramming protocol using the EpiV system. Successful iPSC colonies were obtained. (B) Using the same protocol cells from the adjacent normal tissue from patient samples, AN2 and AN3 were successfully reprogrammed. Distinct iPSC colonies and NANOG positive cells are observed.

Suppl Figure 3;

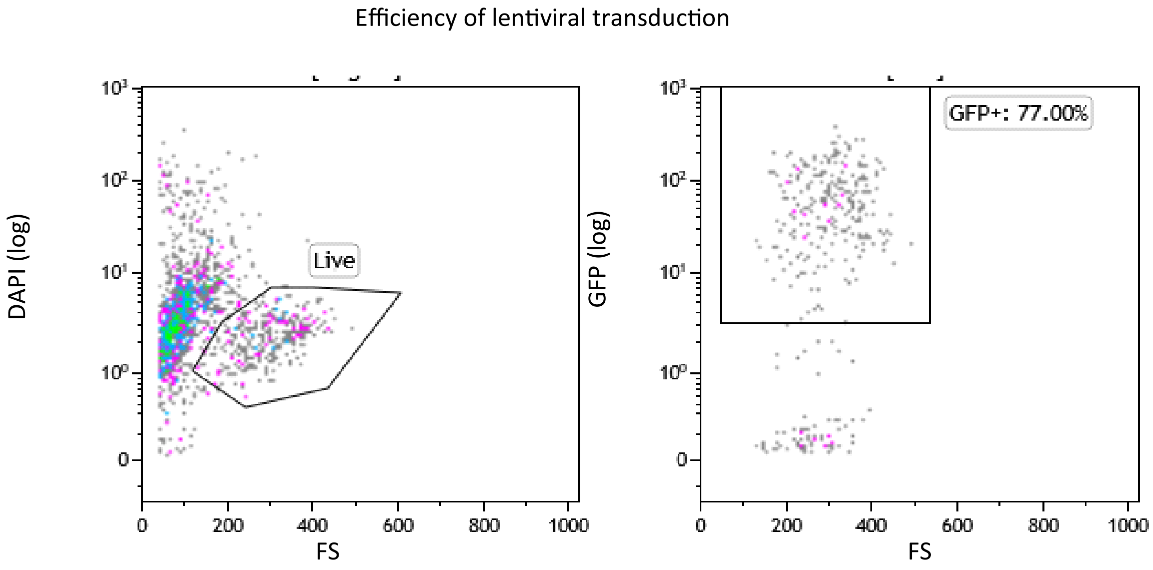

Figure S3. Successful Lentivirus delivery of reprogramming factors to RCC2 patient cells. When the EpiV reprogramming factors failed to reprogram the RCC2 cells, one possibility was that the transfection rate was too low. Lentivirus vectors to deliver the reprogramming factors resulted in 77% of the cells receiving the reprogramming factors.

Supplementary Figure 4

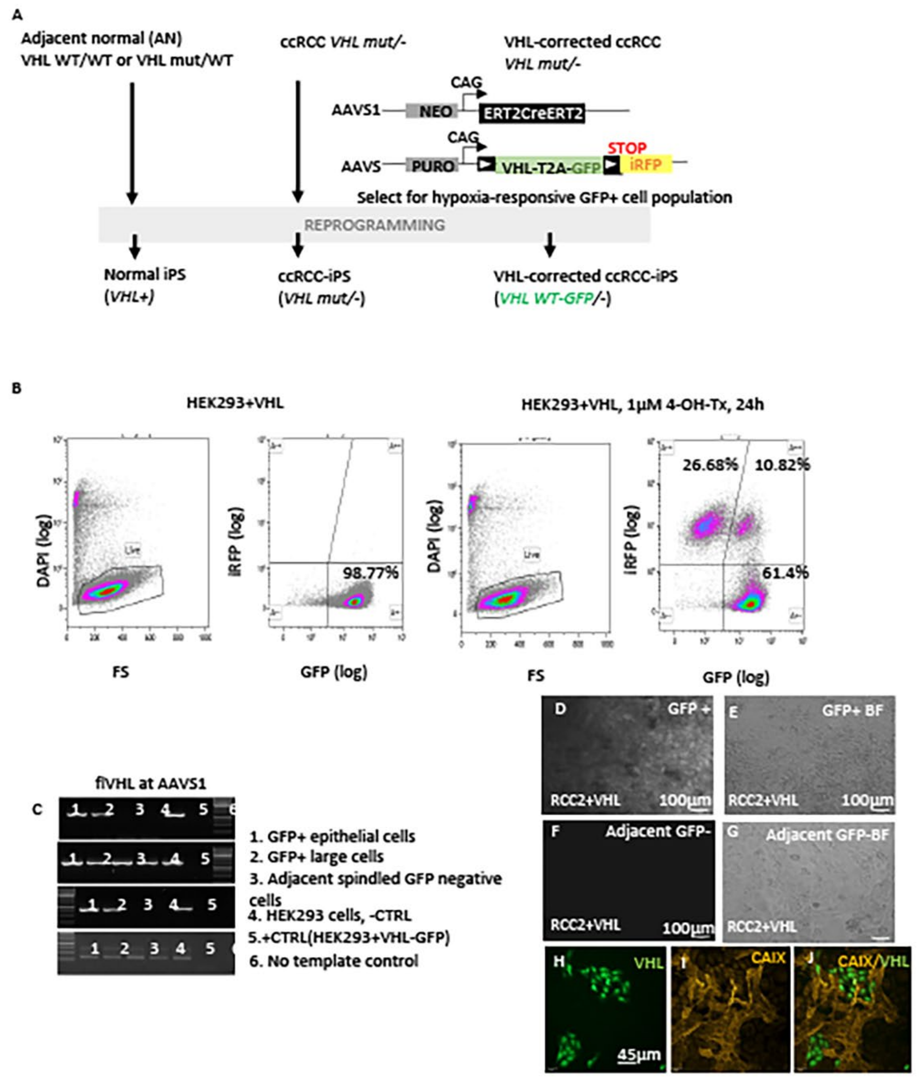

Figure S4. VHL re-expression and reprogramming strategy. (A) The VHL coding sequence was followed by T2A self-cleaving peptide and the Green Fluorescent Protein reporter (GFP), generating both proteins in equivalent amounts. The VHL-T2A-GFP cassette was followed by 'STOP' to avoid the expression of downstream elements. VHL-T2A-GFP-STOP was flanked by loxP sites, the recognition sequence of CRE DNA recombinase. In the presence of CRE, VHL-T2A-GFP is excised, allowing the expression of the red fluorescent reporter iRFP. In our system, the CAG promoter drove continuous expression of a multi-gene co-expression cassette. (B) We selected a 4-hydroxitamoxifen (4-OH-Tx)-inducible variant of CRE (ERT2-Cre-ERT2) to avoid leaky CRE expression and uncontrolled recombination events. The excision of the VHL-T2A-GFP cassette stopped GFP expression and permitted the expression of the iRFP color, thus monitoring the VHL status and allowing the separation of VHL+ and VHL- cells using FACS. HEK293 cells were electroporated with the VHL-T2A-GFP expressing construct to assess tamoxifen responsiveness and efficacy of the GFP-iRFP reporter switch. Over 98% of electroporated HEK293 cells were GFP+, and 26% of the cells completed the GFP/iRFP reporter switch under 1 μM tamoxifen treatment for 24 hours. (C) PCR analysis of transfected and non-transfected cells to confirm the integration of the construct. CAG promoter-loxP-VHL-T2A-GFP-STOP-loxP-iRFP and ERT2-CRE-ERT2 constructs were then co-electroporated with the CRISPR-Cas9 machinery (pXAT plasmid, coding for the AAVS1-targeting sgRNA and Cas9) into RCC2 cells to generate GFP+, VHL-expressing RCC2/VHL-GFP+ cells. (D-G) GFP positive colonies were compact and composed of small epithelioid-like cells, in contrast to the adjacent GFP negative cells which were larger and had loose colonies. (H-J) pVHL expression in the epithelioid colonies was detected by immunocytochemistry, while pVHL-expressing cells showed decreased expression of the hypoxia marker CAIX.

Supplementary Figure 5

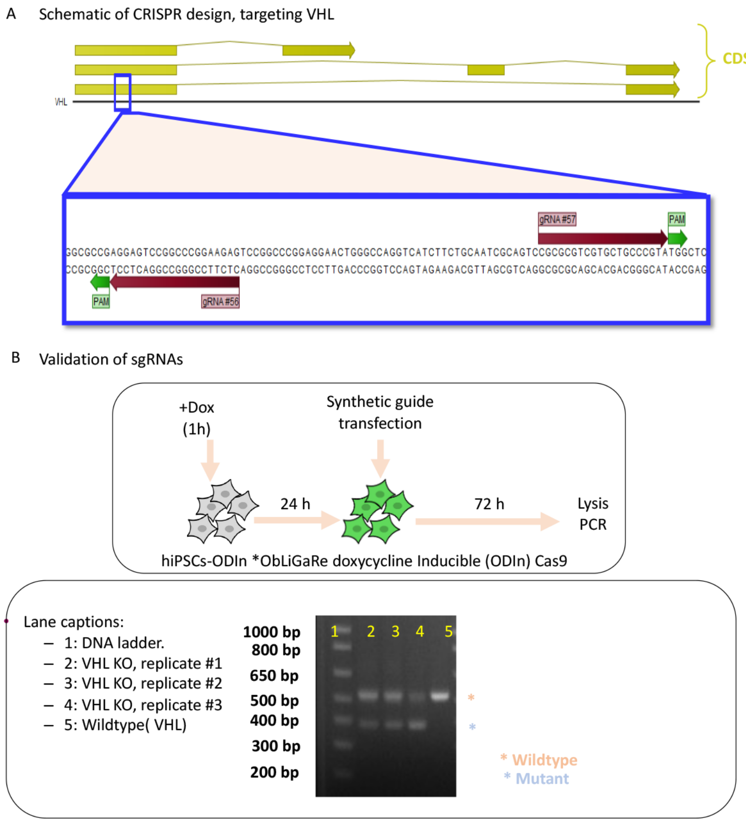

Figure S5. CRISPR design for VHL knock out and validation of edited cells. (A) The first exon was targeted in order remove all three splice variants of VHL. (B) Successful gene targeting confirmed at the genetic level for VHL gene. Replicate # 3 was selected for expansion and cryopreservation. The cells were not clonally selected and therefore contain a mix of wildtype, heterozygous KO and homozygous KO VHL cells.
